# The glycosylated hemoglobin level and the severity of cardiovascular involvement in patients with the first episode of acute coronary syndrome

**DOI:** 10.22088/cjim.15.1.4

**Published:** 2024

**Authors:** Saman Khaleghi, Mohammad ali Bayani, Naghmeh Ziaei, Mohammadtaghi salehiomran, Soraya Khafri

**Affiliations:** 1 Student Research Committee, Babol University of Medical Sciences, Babol, Iran; 2 Clinical Research Development Unite of Rouhani Hospital, Babol University of Medical Sciences, Babol, Iran

**Keywords:** Glycosylated hemoglobin, Coronary atherosclerosis, Acute coronary syndrome.

## Abstract

**Background:**

The current study was carried out aiming at investigating the relationship between glycosylated hemoglobin level and coronary atherosclerosis in patients with the first episode of acute coronary syndrome.

**Methods:**

This case-control study evaluated 450 patients with the first episode of acute coronary syndrome in Ayatollah Rouhani Hospital in Babol (Iran) from 2011 to 2018. Based on glycosylated hemoglobin, patients were divided into three groups of non-diabetic, pre-diabetic, and diabetic (n=150 in each group). Since SYNTAX score and Gensini score are employed to evaluate the extent of cardiovascular disease and predict CVD in patients with CAD over long-term follow-up, we calculated SYNTAX score and Gensini score based on angiographic results.

**Results:**

Concerning the factors related to the severity of cardiovascular involvement, the results revealed no significant difference between the diabetic and pre-diabetic groups in terms of the frequency of patients in terms of SYNTAX score, Gensini score, and the number of vessels involved (0.142 and 87, respectively, and P=0.102). However, this difference between the diabetic and non-diabetic groups, as well as between the pre-diabetic and non-diabetic groups was statistically significant (respectively for SYNTAX score, p< 0.001 and P=0.001; for Gensini score, P=0.013 and P=0.019; and for the number of vessels involved P=0.001and p<0.001).

**Conclusion:**

According to the findings of the current study, since there was no significant difference between diabetic and pre-diabetic patients in terms of the components indicating the severity of cardiovascular involvement, pre-diabetes itself may be associated with the severity of cardiovascular involvement as a predisposing factor.

Acute coronary syndrome (ACS) is associated with a set of cardiac risk factors (1), among which the increased blood lipid levels, blood pressure, abdominal fat, diabetes, smoking, unhealthy lifestyle, and psychosocial factors are modifiable (2, 3). Associated with more severe coronary atherosclerosis, diabetes mellitus is known as a strong risk factor for cardiovascular (CV) diseases (4-6). In Iran, the rate of death due to cardiovascular diseases and diabetes is estimated 400 cases per 10,000 people (7). Patients with both ACS and diabetes are two to four times more exposed to cardiovascular events compared to non-diabetic ACS ones (8). 

Since epidemiological evidence indicates that normal glucose intolerance to overt diabetes may be associated with mortality (9, 10), “pre-diabetes” may be in relationship with an increased risk of coronary artery disease (CAD) (11-14). 

Hemoglobin HbA1c is a form of hemoglobin mainly employed to measure average blood sugar levels over long periods and formed by non-enzymatic glycation through exposure to plasma glucose, measured by examining the component N-[1-deoxy fructosyl] hemoglobin (β-globin) (15). 

In individuals with normal sugar levels, glycosylated hemoglobin has a normal level; however, glycosylated hemoglobin increases as the plasma glucose level rises (16). Besides traditional criteria based on fasting plasma glucose or oral glucose tolerance test (OGTT), the use of hemoglobin A1C (HbA1c) has been approved by The American Diabetes Association (ADA) as a diagnostic test for diabetes mellitus (HbA1c ≥ 6.5%). Increased plasma glucose levels did not only reflect acute stress, but may also be a marker for impaired glucose metabolism, associated with a poorer prognosis in non-diabetic ACS patients (17).

Previous studies have shown the association of high HbA1c in patients with and without diabetes with an increased risk of CAD (18-21); however, other studies have suggested opposite results for the association between HbA1c and ACS, particularly myocardial infarction (22, 23). On the other hand, the association between the level of blood sugar disorder evaluated by HbA1c and cardiac events in diabetic and non-diabetic patients has been specified, but the predictive effect of HbA1c is less known (23). 

Generally, existing studies have presented conflicting results regarding the association between HbA1c at pre-diabetic stage and ACS. Since we assumed that prediabetic patients are at risk of ACS as well as diabetic patients, so the present study was conducted to examine the association between the level of glycosylated hemoglobin in pre-diabetic patients and coronary atherosclerosis in those referred with the first episode of ACS.

## Methods

**Selection of Patients and Study Design: **This cross-sectional study was carried out on consecutive patients with the first episode of ACS referred to Ayatollah Rouhani Hospital in Babol undergoing angiography over the years 2011-2018. The inclusion criteria included patients over 18 years of age diagnosed with ACS according to the guidelines (24) based on electrocardiography criteria and emergency coronary angiography whose HbA1c level was measured. While patients with a history of myocardial infarction and stent implantation or CABG, those with hemoglobinopathy, anemia, reticulosis, a history of blood transfusion and uremia, as well as those with a history of taking diabetic drugs for other reasons, were excluded from the study because of interference with the results. Given the test error (alpha) of 5% and the test power (beta) of 80%, the sample size in each group was considered at least 120 people, and ultimately 150 people were included in each group.

This study was conducted after obtaining the approval of the Research Ethics Committee of Babol University of Medical Sciences with code IR.MUBABOL.HRI.REC.1398.330. According to the American Diabetes Association (ADA) in 2017 (25), patients were assigned to three groups in terms of HbA1c: non-diabetic group (HbA1c less than 5.7%), pre-diabetic group (HbA1c of 5.7-6.4%), and diabetic group (HbA1c equal to and greater than 6.5%).

Other demographic information including age, gender, body mass index (BMI), and clinical information including the results of the performed procedure, the severity of cardiovascular involvement, the course of risk factors for cardiovascular disease including smoking history, dyslipidemia (70 mg/dL ≤ LDL), family history of CAD (relatives before the age of 55 for men and 65 for women), and hypertension (systolic blood pressure over or equal to 140 mmHg, diastolic blood pressure over or equal to 90 mmHg, and/or the use of antihypertensive treatments) were collected. BMI was calculated using the formula of weight divided by the second power of height (kg/m^2^) and all patients with a BMI over or equal to 25 kg/m^2 ^were considered obese (26).

In the first morning after admission, one cc of completely citrated peripheral venous blood was taken from all patients from the antecubital vein of the patients to determine the HbA1c and other biochemical parameters and sent to the hospital laboratory. The level of HbA1c was measured through immune-turbidimetric assay in COBAS INTEGRA 400 device (Roche Diagnostics, Mannhein, Germany).

ACS includes unstable angina, NSTEMI (non-ST segment elevation MI) myocardial infarction and STEMI (St Segment elevation MI) myocardial infarction. After the diagnosis of ACS, coronary angiography was carried out at the same admission by standard Judkins or radial methods. Each coronary artery was displayed in at least two various planes. All coronary angiograms were recorded in the system in DICOM format.

Percutaneous coronary intervention (PCI) procedures were performed by standard techniques. Based on angiography, cardiovascular disease was defined with coronary stenosis greater than 50% of the luminal diameter. The extent of CAD was classified as: SVD: single-vessel disease, 2VD: two-vessel disease, and 3VD: three-vessel disease

SYNTAX and Gensini scores in combination with clinical variables were utilized to assess the extent of CAD and forecast CV over long-term follow-up in patients with CAD (14). We calculated the SYNTAX and Gensini scores according to the cardiovascular findings entered in www.syntaxscore.com. Based on the SYNTAX score, patients were assigned to three categories (21): Low SXScore group (22 ≥ SXScore), medium SXScore group (SXScore of 23-32), and high SXScore group (33 ≤ SXScore).

Besides, the Gensini score is equal to the sum of all sections’ scores (the score of each section is equal to the section’s weighting factor multiplied by a severity score). As explained previously, the weighting factors of the section are between 0.5 and 5.0. Severity scores reflect the specific percentage of diameter reduction in the cardiovascular segment for 100%, 99%, 90%, 75%, and 25% respectively as 32, 16, 8, 4, 2, and 1. The scores of 22 < SYNTAX and 20 < Gensini were defined as high scores (20).

The key difference between the two scores was that the stenosis in Gensini score starts at 25% and in SYNTAX at 50%; moreover, in a patient with STEMI, Gensini was calculated after PCI without inclusion of the culprit lesion and in patients with STEMI, the SYNTAX score of the culprit lesion was calculated before stent implantation (11, 15). All measurements were conducted by a cardiologist blinded to the clinical conditions and their tests’ status.

**Statistical Analysis:** The data were analyzed using SPSS 22 software. Kolmogorov-Smirnov test was employed to check the data normality. Quantitative data were reported as mean and standard deviation (SD). The mean of mortality, in-hospital complications, and the severity of cardiovascular involvement in patients were compared by ANOVA test and according to the classification of patients based on HBA1C. Correlation between variables was investigated using non-parametric Spearman test. P-values less than 0.05 were considered as a significant level in all cases.

## Results

In the current study, 150 patients were included in each group and in terms of age and gender, no significant difference was found between the three groups. The patients’ basic information is exhibited in table 1, and as observed, in the non-diabetic patients’ group, 101 (67.3%) had dyslipidemia and 100 (66.6%) had hypertension. While in the pre-diabetes and diabetic patients’ groups, respectively 97 (64.7%) people and 104 (69.3%) people had dyslipidemia, and 98 (65.3%) people and 112 (74.6%) people had hypertension. No significant difference was observed between the three groups in terms of dyslipidemia and hypertension (P=0.624 and P=0.804, respectively). 

**Table 1:** Comparison of basic information in non-diabetic, pre-diabetic, and diabetic ACS patients

		**Non-diabetic**	**Pre-diabetic**	**Diabetic**	**P-value** **between 3 groups**
**Gender (n)**	**Females**	80	76	77	0.645
	**Males**	70	74	73	
**Age (mean ** **±** ** SD (year))**		59.00 (5.56)	57.38 (11.41)	58.06 (11.49)	0.363
**Dyslipidemia (n)**	**Yes**	101	97	104	0.624
	**No**	49	53	46	
**Hypertension (n)**	**Yes**	100	98	112	0.804
	**No**	50	52	38	

Concerning the factors related to the severity of cardiovascular involvement, a comparison was made among the three non-diabetic, pre-diabetic, and diabetic groups. As observed in table 2, there was a significant difference among the three patients’ groups in terms of frequency and according to SYNTAX and Gensini criteria, as well as the number of vessels involved so that frequency in all three variables had no significant difference between the diabetic and pre-diabetic groups. However, there was a statistically significant difference between the diabetic group and non-diabetic patients, as well as between the pre-diabetic group and non-diabetic people. Table 2 shows their significance results. The present research also investigated other variables affecting the SYNTAX score so that a significant association was found between the presence of hypertension, age, and the SYNTAX score (both P < 0.001). Nevertheless, no significant relationship between SYNTAX score, gender, and dyslipidemia was observed (P = 0.589 and 0.289, respectively). Furthermore, variables affecting the Gensini score were examined, revealing a significant relationship between the presence of hypertension, age, and the Gensini score (both P < 0.001). Nevertheless, no significant relationship between the Gensini score with gender and dyslipidemia was observed (P=0.532 and 0.937, respectively).

Investigation of the number of vessels involved in angiography revealed that the patients’ average age as well as hypertension have a significant relationship with the number of vessels involved (both p < 0.001). However, no significant relationship between the number of vessels involved, gender, and dyslipidemia was observed (P=0.410 and 0.155, respectively).

**Table 2:** Comparison of factors describing the severity of cardiovascular involvement in non-diabetic, pre-diabetic, and diabetic ACS patients

		**Groups**			**P-value**			
		**Non-diabetic**	**Pre-diabetic**	**Diabetic**	**P-value** **between 3 groups**	**P-value** **Diabetes** **Versus** **Pre-diabetes**	**P-value** **Diabetes** **Versus** **Normal control**	**P-value** **pre-diabetes Versus** **Normal control**
**SYNTAX score**	**Low risk**	(80.6%) 121	(60.7%) 91	(48.7%) 73	0.002*	0.142	< 0.001*	0.001*
	**Medium risk**	(5.4%) 8	(22.7%) 34	(26%) 39				
	**High risk**	(14%) 21	(16.7%) 25	(25.3%) 38				
**Gensini score**	**Low risk**	(92.6%) 139	(83.3%) 122	(57.3%) 86	0.027*	0.870	0.013*	0.019*
	**High risk**	(7.4%) 11	(18.7%) 28	(42.7%) 64				
**Number of vessels involved**	**SVD**	(80%) 120	(54.7%) 82	(48.7%) 73	< 0.001*	0.102	0.001*	< 0.001*
	**2VD**	(6%) 9	(30.7%) 46	(26%) 39				
	**3VD**	(14%) 21	(14.6%) 22	(25.3%) 38				

* Significant relationship with 95% confidence level

## Discussion

This retrospective cross-sectional study was carried out aiming at examining the relationship between the percentage of glycosylated hemoglobin and the severity of cardiovascular involvement in patients with the first episode of ACS and revealed the higher cardiovascular involvement based on the SYNTAX and Gensini scores and in terms of the number of vessels involved in diabetic and pre-diabetic patients than in normal patients.

Pre-diabetes refers to an intermediate stage of blood sugar control with glycemic parameters higher than normal and lower than the diabetic threshold. Under these conditions, the patient is exposed to a high risk of becoming diabetic (5-10% every year) and this is associated with complications of diabetes, including cardiovascular complications (19). Our study findings are supported by previous observations reporting that based on both the SYNTAX and Gensini criteria, the percentage of glycosylated hemoglobin is associated with the severity of cardiovascular involvement (18, 27). Moreover, a similar recent study conducted by Mirza et al. in the Middle East supports our findings about the population living in this region (28). This study used the SYNTAX scoring scale to assess the cardiovascular involvement level, showing a positive linear relationship between the percentage of glycosylated hemoglobin and the severity of CAD in non-diabetic and pre-diabetic patients’ angiography so that, the group with higher HbA1c% was associated with the larger number of vessels involved and the higher severity of the involvement. This study used logistic regression to determine the independent predictor variable and HbA1c% was the only independent variable among common risk factors. All angiographic parameters in comparing diabetic and pre-diabetic patients showed higher severity of disease in pre-diabetic patients. Also, in another study carried out by Ahmed et al., it similarly revealed the higher number of diabetic and pre-diabetic patients than non-diabetic patients with multi-vessel involvement in angiography (29).

In Acar et al.’s study, examining the relationship between pre-diabetes and the progression of coronary atherosclerosis among patients with the first episode of ACS, similar to our study, SYNTAX and Gensini scores, and 3VD levels were significantly higher in pre-diabetic patients compared to the non-diabetic patients. However, this rate was not significantly different between pre-diabetic and diabetic patients (22).

Although the present study did not investigate the prognosis of HbA1c in the studied group, the findings related to the higher SYNTAX and Gensini scores may indicate a worse prognosis. In other words, the prognosis can be assumed worse in the pre-diabetes and diabetes groups with higher HbA1c. Moreover, there are several meta-analyses supporting this. These studies showed an increased risk of mortality in non-diabetic patients with high HbA1c (30-32). Recently, several other studies revealed the higher rate of in-hospital mortality and long-term mortality in the pre-diabetic and diabetic patients’ groups than that in the non-diabetic group (29, 33-35). A more interesting finding in Lior Lupu et al.’s study was that the mortality rate in the pre-diabetic group was higher than that in the diabetic group (34). This finding was similarly observed in Yahyavi et al.’s study; so that a 12-month follow-up among outpatients showed the highest adverse cardiovascular events in people with HbA1c just below the threshold of diabetes, i.e. pre-diabetes (35).

In spite of the strong relationship between pre-diabetes and adverse cardiovascular outcomes, the recommended treatment still focuses on lifestyle changes, suggesting pharmacotherapy with metformin only (34). In Yahyavi et al.’s study(35), where the rate of adverse cardiovascular events was the highest among pre-diabetic patients, a lower incidence of starting to take cardioprotective drugs and hypoglycemic drugs was indicated in this group of patients. This finding can explain why these patients are at higher mortality risk. In addition, another study by Kim et al. revealed that pre-diabetes status was a key predictor of in-hospital mortality in patients with acute ischemic stroke (36). Nevertheless, before hospitalization, the pre-diabetic patients have been treated with statins and antiplatelet drugs significantly less than the diabetic patients that can be another justification for the point that due to their cardiovascular risk, pre-diabetic patients may not benefit from appropriate medical measures. In this regard, Gholap et al. in their study recommended that patients with ACS and no previous history but with diabetes symptoms and with HbA1c higher than 6.5% must be treated as a person with diabetes (37).

Given the recent studies and what was mentioned, it was shown that coronary atherosclerosis in diabetic patients occurs more severely than in normal people (38, 39), being often diagnosed at advanced stages (38), while cardiovascular disease, particularly in patients with ACS is either asymptomatic or diagnosed late at pre-diabetes stages (13, 40, 41). Accordingly, Nanayakkara et al. in their study suggested that HbA1c be controlled in every ACS patient without previous history of diabetes (42). In general, the present study revealed that based on SYNTAX and Gensini scoring criteria, the increased HbA1c value has a strong association with the severity of cardiovascular involvement in angiography. In accordance with the findings of this study, besides what was obtained from other studies (28, 30, 31, 43), measuring HbA1c along with investigation other risk factors of cardiovascular disease in any patient with ACS without a history of diabetes may be considered beneficial. The results of this study generally support the previous findings in which pre-diabetes status has been considered as a key factor for adverse cardiovascular complications and mortality, indicating the need for preventive strategies to prevent cardiovascular events in patients with diabetes and pre-diabetes.

The present study was also faced with limitations, among which the single-centered nature can be mentioned. Furthermore, in this study, only patients with ACS were included; it is recommended to investigate patients with cardiovascular disease with other manifestations as well. Its cross-sectional nature as well as the lack of long-term follow-up to investigate the outcome of the treatment in patients was another limitation of this study.
